# Meteorological Observations for June, 1857, at Atlanta, Ga.

**Published:** 1857-07

**Authors:** J. G. Westmoreland


### METEOROLOGICAL OBSERVATIONS FOR JUNE, 1857, AT ATLANTA, GA.

| JUNE. | THERMOMETER. | | | BAROMETER. | | | WIND. | REMARKS. |
|---|---|---|---|---|---|---|---|---|
| | 7 A. M. | 2 P. M. | 7 P. M. | 7 A. M. | 2 P. M. | 7 P. M. | | |
| 1 | 64 | 60 | 62 | 29.90 | 29.85 | 29.85 | | Cloudy $\frac{5}{8}$. |
| 2 | 62 | 68 | 68 | 29.85 | 29.95 | 29.95 | N. W. | Fair. |
| 3 | 58 | 78 | 60 | 29.92 | 30.05 | 30. | W. | Fair. |
| 4 | 58 | 86 | 74 | 30.05 | 30.07 | 30. | N. E. | Fair. |
| 5 | 64 | 84 | 68 | 29.95 | 30. | 29.95 | W. | Cloudy—Rain $\frac{3}{8}$. |
| 6 | 60 | 70 | 60 | 29.95 | 30. | 30. | N. E. | Cloudy. |
| 7 | 60 | 84 | 82 | 29.95 | 30.05 | 30. | E. | Hazy. |
| 8 | 74 | 90 | 84 | 30.10 | 30.12 | 30.12 | S. E. | Fair. |
| 9 | 78 | 96 | 68 | 30.20 | 30.20 | 30.10 | S. W. | Fair. |
| 10 | 70 | 90 | 74 | 30.05 | 30.05 | 29.92 | S. W. | Cloudy. |
| 11 | 68 | 86 | 76 | 29.80 | 29.80 | 29.83 | N. W. | Fair. |
| 12 | 62 | 88 | 80 | 29.95 | 30. | 30. | W. | Fair. |
| 13 | 68 | 94 | 82 | 30. | 30.15 | 30.10 | W. | Fair. |
| 14 | 74 | 88 | 88 | 30.12 | 30.20 | 30.18 | S. W. | Fair. |
| 15 | 80 | 72 | 64 | 30.20 | 30.15 | 30.10 | N. W. | Cloudy—Rain 1-16. |
| 16 | 68 | 88 | 76 | 30.08 | 30.12 | 30.05 | N. W. | Cloudy. |
| 17 | 68 | 74 | 68 | 30. | 29.95 | 29.95 | W. | Cloudy—Rain $\frac{3}{4}$. |
| 18 | 60 | 80 | 74 | 29.90 | 29.95 | 29.90 | W. | Hazy—Drizzly. |
| 19 | 56 | 75 | 66 | 29.85 | 29.95 | 29.95 | N. W. | Fair. |
| 20 | 64 | 82 | 78 | 29.95 | 30. | 30.05 | W. | Fair. |
| 21 | 76 | 88 | 78 | 30.10 | 30.10 | 30.05 | S. E. | Fair. |
| 22 | 68 | 78 | 64 | 30.05 | 30.10 | 30.02 | N. W. | Fair. |
| 23 | 58 | 80 | 70 | 30. | 30.12 | 30.05 | N. W. | Fair. |
| 24 | 64 | 78 | 70 | 30.10 | 30.12 | 30.10 | E. | Hazy. |
| 25 | 72 | 82 | 77 | 30.12 | 30.12 | 30.10 | S. E. | Hazy. |
| 26 | 70 | 88 | 75 | 30.10 | 30.15 | 30.10 | S. | Cloudy—Rain $\frac{1}{8}$. |
| 27 | 70 | 86 | 72 | 30.10 | 30.15 | 30.05 | S. W. | Cloudy—Rain $\frac{1}{8}$. |
| 28 | 70 | 80 | 76 | 30.05 | 30.05 | 30. | W. | Hazy. |
| 29 | 76 | 84 | 76 | 30. | 30. | 29.95 | N. W. | Fair. |
| 30 | 76 | 80 | 72 | 30. | 29.95 | 29.90 | N. W. | Fair. |

Furnished by

J. G. WESTMORELAND, M. D.

